# Evidence That Acetylsalicylic Acid Attenuates Inflammation in the Walls of Human Cerebral Aneurysms: Preliminary Results

**DOI:** 10.1161/JAHA.112.000019

**Published:** 2013-02-22

**Authors:** David M. Hasan, Nohra Chalouhi, Pascal Jabbour, Aaron S. Dumont, David K. Kung, Vincent A. Magnotta, William L. Young, Tomoki Hashimoto, H. Richard Winn, Donald Heistad

**Affiliations:** 1 Department of Neurosurgery, Carver College of Medicine, University of Iowa, Iowa City, IA (D.M.H., D.K.K.); 2 Department of Neurosurgery, Thomas Jefferson University and Jefferson Hospital for Neuroscience, Philadelphia, PA (N.C., P.J., A.S.D.); 3 Department of Radiology, Carver College of Medicine, University of Iowa, Iowa City, IA (V.A.M.); 4 Department of Neurological Surgery, University of California San Francisco, San Francisco, CA (W.L.Y.); 5 Department of Anesthesia and Perioperative Care, University of California San Francisco, San Francisco, CA (W.L.Y., T.H.); 6 Department of Neurosurgery, Hofstra University and Lenox Hill Hospital, New York City, NY (R.W.); 7 Departments of Internal Medicine and Pharmacology, Carver College of Medicine, University of Iowa, Iowa City, IA (D.H.)

**Keywords:** aneurysm, aspirin, ferumoxytol, inflammation

## Abstract

**Background:**

Inflammatory cells and molecules may play a critical role in formation and rupture of cerebral aneurysms. Recently, an epidemiologic study reported that acetylsalicylic acid (ASA) decreases the risk of aneurysm rupture. The goal of this study was to determine the effects of ASA on inflammatory cells and molecules in the walls of human cerebral aneurysms, using radiographic and histological techniques.

**Methods and Results:**

Eleven prospectively enrolled patients harboring unruptured intracranial aneurysms were randomized into an ASA‐treated (81 mg daily) group (n=6) and an untreated (control) group (n=5). Aneurysms were imaged at baseline using ferumoxytol‐enhanced MRI to estimate uptake by macrophages. After 3 months, patients were reimaged before undergoing microsurgical clipping. Aneurysm tissues were collected for immunostaining with monoclonal antibodies for cyclooxygenase‐1 (COX‐1), cyclooxygenase‐2 (COX‐2), microsomal prostaglandin E2 synthase‐1 (mPGES‐1), and macrophages. A decrease in signal intensity on ferumoxytol‐enhanced MRI was observed after 3 months of ASA treatment. Expression of COX‐2 (but not COX‐1), mPGES‐1, and macrophages was lower in the ASA group than in the control group.

**Conclusions:**

This study provides preliminary radiographical and histological evidence that ASA may attenuate the inflammatory process in the walls of human cerebral aneurysms.

**Clinical Trial Registration:**

URL: http://www.clinicaltrials.gov. Unique identifier: NCT01710072.

## Introduction

Evidence from studies in humans and experimental animals suggests that inflammation is a key mechanism in formation and rupture of cerebral aneurysms.^1–10^ Several inflammatory mediators, including cyclooxygenase‐2 (COX‐2) and microsomal prostaglandin E2 synthase‐1 (mPGES‐1), are upregulated in the walls of cerebral aneurysms.^3^ We have demonstrated recently that COX‐2 and mPGES‐1 are expressed in the walls of human cerebral aneurysms.^11^ These inflammatory molecules were more abundantly expressed in ruptured than in unruptured aneurysms.^11^

Macrophages appear to play a key role in aneurysm formation, as macrophage depletion reduces the prevalence of cerebral aneurysms in mice.^8^ We have demonstrated the feasibility of imaging macrophages within the walls of human cerebral aneurysms using ferumoxytol‐enhanced MRI.^12^

In a secondary analysis (case–control study) of the International Study of Unruptured Intracranial Aneurysms (ISUIA), we reported that daily intake of acetylsalicylic acid (ASA) reduces the incidence of rupture of cerebral aneurysms by 60%.^13^ The goal of the present study was to test the hypothesis that ASA treatment reduces the inflammatory response in the walls of human cerebral aneurysms. We used ferumoxytol‐enhanced MRI to estimate uptake of the tracer by macrophages and immunostaining to examine expression of macrophages and inflammatory molecules in aneurysms.

## Material and Methods

### Study Population

This trial was approved by the University of Iowa Institutional Review Board. All enrolled patients gave written informed consent to participate in the study.

Eleven consecutive patients with incidentally discovered saccular intracranial aneurysms presenting to the Neurosurgery Department at the University of Iowa Hospitals and Clinics were prospectively enrolled in the study between January 2011 and June 2012. The patients chose to proceed with aneurysm treatment after discussion with their physicians. The aneurysms were not suitable for endovascular treatment, as deemed by the first author (D.H.), who is a neurosurgeon dually trained in both endovascular and microsurgical treatment of aneurysms. Microsurgical clipping of the aneurysm was recommended to these patients.

Adult patients (age >18 years) were considered eligible for the study. Exclusion criteria were (1) pregnant women; (2) having a history of allergy or hypersensitivity to iron, dextran, or iron‐polysaccharide preparations; (3) patients requiring monitored anesthesia or IV sedation for MRI; (4) patients with contraindication to MRI; (5) having renal insufficiency, hepatic insufficiency, or iron overload; (6) patients receiving combination antiretroviral therapy (which is a contraindication to ferumoxytol infusion); (7) patients already taking aspirin; and (8) having a contraindication to aspirin use.

Eleven patients harboring 12 aneurysms were enrolled and randomized into an ASA‐treated group (n=6) and an untreated (control) group (n=5). Assignment of patients to either group was made randomly in clinic, using an envelope containing a paper assigning either to aspirin or no aspirin. The patients were not matched for age, race, sex, smoking, or other comorbidities because of the small size of the cohort. Patients in the ASA group received ASA (81 mg) daily for 3 months. Patients in the control group did not receive a placebo. Aneurysms were imaged using T2* gradient‐echo (TE=20 ms, TR=500 ms, flip angle=20°, FOV=220×220, matrix=512×384, bandwidth=260 Hz/pixel, averages=1, slice thickness/gap=3.0/0.3 mm) and T1‐weighted spin‐echo (TE=2.6 ms, TR=317 ms, flip angle=70°, FOV=220×220, matrix=384×307, bandwidth=330 Hz/pixel, averages=2, slice thickness/gap=3.0/0.3 mm) imaging. Patients were imaged at baseline and immediately and 72 hours after ferumoxytol infusion. Subtraction images were obtained (immediately postinfusion minus preinfusion and 72 hours postinfusion minus preinfusion). Patients were then placed on ASA or served as untreated controls for 3 months. After 3 months, imaging was performed only 72 hours after ferumoxytol infusion. We have previously demonstrated that the optimal timing for macrophage imaging in the walls of human cerebral aneurysms is 72 hours postferumoxytol infusion.^12^ Seventy‐two‐hour postinfusion images at baseline and after 3 months were coregistered and then subtracted (72 hours postinfusion at baseline minus 72 hours postinfusion after 3 months).

Patients subsequently underwent microsurgical clipping for treatment of their aneurysms. ASA was stopped 1 day before the procedure. Aneurysm tissues were collected for immunostaining with monoclonal antibodies.

### Analysis of Aneurysm Dome Tissue

Tissues were immunostained with monoclonal antibodies to COX‐1 (Epitomics, Burlington, CA), COX‐2 (Epitomics, Burlington, CA), and mPGES‐1 (Cayman Chemical, Ann Arbor, MI) and macrophages using monoclonal antibodies to CD 68+ (ABCAM, Cambridge, MA).

Assessment of slides stained only for COX‐1, COX‐2, mPGES‐1, and macrophages was made by an observer who was not aware of the source of tissues or clinical data.

Semiquantitative analysis of the slides was performed as described previously^11^ based on manual cell count (immunostain‐positive cells) per high‐power field (HPF; 40×): grade 0, 0 cells per HPF; grade 1, 1 to 10 cells per HPF; grade 2, 11 to 20 cells per HPF; and grade 3, >20 cells per HPF.

### Imaging of Aneurysms

Ferumoxytol 5 mg/kg at a dilution of 30 mg/mL was administered as a 1‐time dose to all patients enrolled in the study. The off‐label use of the drug in a research protocol was approved by the institutional review boards at the University of Iowa, and patients were monitored for adverse reactions to ferumoxytol infusion. All MRIs were completed on a Siemens 3T TIM Trio system. Two neuroradiologists independently reviewed MRI images from all patients in both groups in a blinded fashion and rated the change in loss of signal intensity. On the basis of the uptake of ferumoxytol nanoparticles by macrophages localized in the walls of human cerebral aneurysms and the change in MRI signal corresponding to this uptake, inflammation in the walls of aneurysms was classified as “attenuated” if the MRI signal changes decreased in the follow‐up images or “stable” if the MRI signal did not change in the follow‐up imaging studies.

Aneurysm size was measured using CT angiogram (CTA), magnetic resonance angiogram (MRA), and/or digital subtraction diagnostic angiogram. Statistical analysis was performed using the Mann–Whitney test. *P*<0.05 was considered statistically significant. The kappa test was used to measure interrater agreement between the 2 neuroradiologists.

## Results

Eleven patients harboring 12 aneurysms were enrolled in the study. The average age of patients was 60.1 years (range, 45 to 70 years) in the ASA group and 54.4 years (range, 47 to 67 years) in the control group. Mean aneurysm size (using the larger diameter) was 8.4 mm (range, 5 to 12 mm) in the ASA group and 9.2 mm (range, 5 to 14 mm) in the control group. No adverse events related to ASA treatment or ferumoxytol infusion were noted. Table 1 summarizes patient demographics and aneurysm characteristics.

**Table 1.** Patient and Aneurysm Characteristics

Patient No.	Age	Sex	Aneurysm Location	Aneurysm Size (mm)	Aspirin Treatment
1	50	F	L‐ICA	9×6	No
2	56	M	R‐MCA	5×4	No
3	52	F	L‐MCA	10×10	No
4	67	F	Basilar tip	7×8	No
5	47	M	R‐Pcomm	14×11	No
6	70	F	L‐MCA	4×5	Yes
			Basilar tip	6×6	Yes
7	69	F	Acomm	7×5	Yes
8	45	F	R‐Ophthalmic	12×9	Yes
9	49	F	L‐MCA	7×5	Yes
10	59	F	R‐PICA	9×11	Yes
11	69	F	R‐Posterior carotid wall	11×7	Yes

F indicates female; M, male; L, left; R, right; ICA, internal carotid artery; MCA, middle cerebral artery; Pcomm, posterior communicating artery; Acomm, anterior communicating artery; PICA, posterior communicating artery.

### Imaging Findings

**Figure 1.**
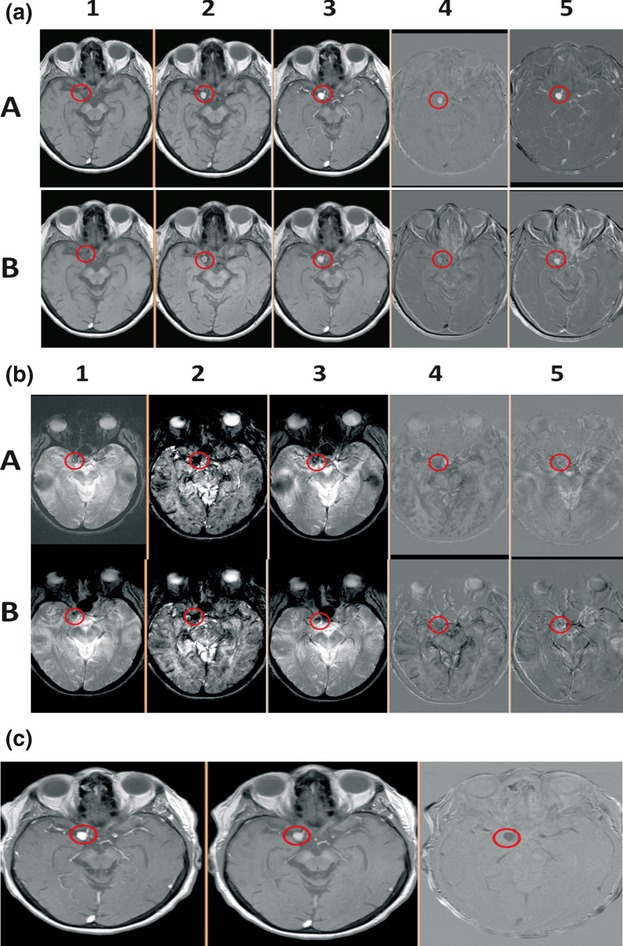
Right posterior carotid wall aneurysm from a 69‐year‐old female patient. (a) T1 spin‐echo sequence. Images A1 to A5 are baseline images (ie, before aspirin treatment). A1, Before ferumoxytol infusion. A2, Immediately after ferumoxytol infusion. A3, Seventy‐two hours after ferumoxytol infusion. A4, A2 minus A1. A5, A3 minus A1. Images B1 to B5 were obtained after 3 months of aspirin treatment. B1, Before ferumoxytol infusion. B2, Immediately after ferumoxytol infusion. B3, Seventy‐two hours after ferumoxytol infusion. B4, B2 minus B1. B5, B3 minus B1. (b) T2* sequence. Images A1 to A5 are baseline images (ie, before aspirin treatment). A1, Before ferumoxytol infusion. A2, Immediately after ferumoxytol infusion. A3, Seventy‐two hours after ferumoxytol infusion. A4, A2 minus A1. A5, A3 minus A1. Images B1 to B5 were obtained after 3 months of aspirin treatment. B1, Before ferumoxytol infusion. B2, Immediately after ferumoxytol infusion. B3, 72 hours after ferumoxytol infusion. B4, B2 minus B1. B5, B3 minus B1. (c) T1 spin‐echo sequence. Left, baseline image (ie, before aspirin treatment) 72 hours after ferumoxytol infusion; middle, 3‐month follow‐up images (after aspirin treatment) obtained 72 hours following ferumoxytol infusion; right, A2 minus A1. Note the decrease in signal intensity on various magnetic resonance imaging (MRI) sequences after aspirin treatment.

Seven aneurysms (in 6 patients) in the ASA group and 5 aneurysms (in 5 patients) in the control group were imaged and analyzed. The signal intensity in the ASA group was decreased in the walls of cerebral aneurysms on T2*GE and postferumoxytol T1 sequences after 3 months of ASA treatment (Figure 1). In the control group, signal intensity on both T2* gradient‐echo and postferumoxytol T1 sequences did not change after 3 months of observation. Thus, inflammation in aneurysm walls, reflected by tracer uptake by macrophages, was attenuated in the ASA group and stable in the control group. The percentage of agreement about signal change between the 2 neuroradiologists, measured using the kappa estimate of agreement,^14^ was 100%.

### Histological Findings

**Figure 2.**
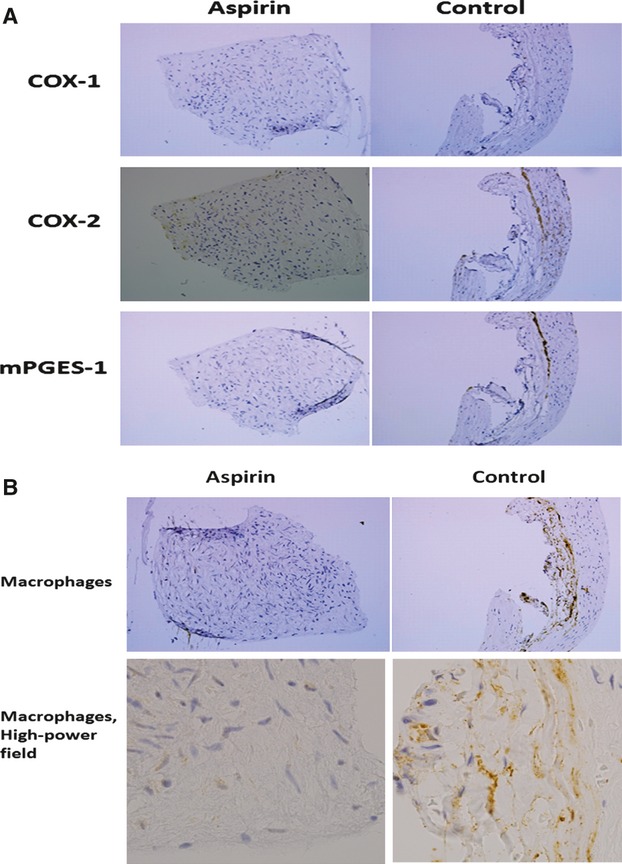
A and B, Immunostaining for 2 aneurysms, 1 from the ASA group and the other from the control group. A, Immunostaining for COX‐1 is similar between the 2 aneurysms. Immunostaining shows downregulation of COX‐2 and mPGES‐1 in the ASA group compared with the control group. B, Immunostaining shows downregulation of macrophages in the ASA group compared with the control group. ASA indicates acetylsalicylic acid; COX‐1, oxygenase‐1; COX‐2, cyclooxygenase‐2; mPGES‐1, microsomal prostaglandin E2 synthase‐1.

**Figure 3.**
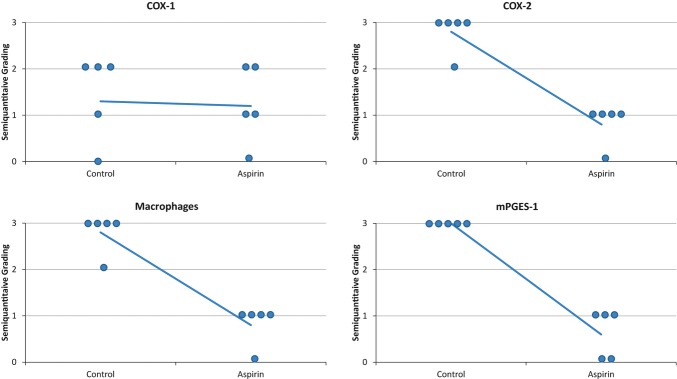
Semiquantitative grading for aneurysm tissues collected from 5 aneurysms in the ASA group and 5 aneurysms in the control group. Immunostaining for COX‐1 is similar in both groups. Immunostaining shows downregulation of COX‐2, mPGES‐1 and macrophages in the ASA group. ASA indicates acetylsalicylic acid; COX‐1, cyclooxygenase‐1; COX‐2, cyclooxygenase‐2; mPGES‐1, microsomal prostaglandin E2 synthase‐1.

In the patient harboring 2 aneurysms, only 1 aneurysm was clipped. Patient 11 received aspirin treatment but did not undergo surgical clipping. Thus, aneurysm tissue was analyzed in 10 patients: 5 in the ASA group and 5 in the control group. Immunostaining with COX‐1 was similar in the 2 groups of patients. There was decreased expression of COX‐2 (median, 24 versus 5 cells per 40× HPF), mPGES‐1 (median, 33 versus 4 cells per 40× HPF), and macrophages (median, 26 versus 5 cells per 40× HPF) in the ASA group (Table 2 and Figures 2 and 3) compared with the control group (*P*<0.05).

**Table 2.** Absolute Count of Inflammatory Molecules and Cells

	COX‐1	COX‐2	mPGES‐1	Macrophage
Control group	0	11	24	12
	9	22	31	21
	12	30	34	33
	14	27	37	35
	17	29	39	27
ASA group	0	0	0	0
	5	3	0	5
	9	7	5	6
	13	9	9	8
	14	6	8	4

COX‐1 indicates cyclooxygenase‐1; COX‐2, cyclooxygenase‐2; mPGES‐1, microsomal prostaglandin E2 synthase‐1; ASA, acetylsalicylic acid.

## Discussion

Inflammation is a key mechanism in the formation and rupture of intracranial aneurysms.^1–10^ Our previous work demonstrated that ASA intake is associated with a decreased risk of aneurysm rupture by as much as 60%.^13^ We speculated that this intriguing and somewhat counterintuitive finding may be related to anti‐inflammatory effects of ASA.^15^ Indeed, we found in a recent study that proinflammatory molecules COX‐2 and mPGES‐1 are expressed in higher levels in the walls of ruptured compared with unruptured human cerebral aneurysms.^11^ In light of these findings, we speculated that protection by ASA against rupture of aneurysms may be mediated in part by inhibition of COX‐2/mPGES‐1.

The major conclusion of this study is that, based on findings in a small number of patients, ASA treatment may attenuate the inflammatory response in the walls of unruptured human cerebral aneurysms. Effects of ASA on the inflammatory response in the walls of cerebral aneurysms were assessed using ferumoxytol‐enhanced MRI. This is a noninvasive imaging method that detects inflammation in the aneurysm wall, using macrophage uptake of ferumoxytol as a surrogate marker.^12^ The MRI signal was stable in the control group and decreased in the ASA group. Thus, uptake of ferumoxytol nanoparticles by macrophages decreased with ASA treatment, presumably from attenuation of inflammation in aneurysm walls. We also observed histologically a decrease in macrophages and expression of COX‐2/mPGES‐1 in aneurysms from patients treated with ASA. As discussed above, macrophages and COX‐2/mPGES‐1 are thought to be pivotal in the formation and rupture of cerebral aneurysms.^1,3,5,8,16–17^

### Inflammation and Cerebral Aneurysms

Several experimental and human studies have clarified the pathophysiology of aneurysm formation and rupture. Low shear stress elicits an inflammatory response in arterial walls.^5,17–18^ Endothelial dysfunction with activation of proinflammatory and proliferative pathways is an integral part of the inflammatory response and involves activation of nuclear factor kappa B (NF‐kB),^2^ monocyte chemoattractant protein 1,^1,19^ and vascular cell adhesion molecule 1,^10,20^ among other inflammatory mediators. Subsequently, monocytes infiltrate the aneurysm wall, undergo activation, and secrete several cytokines that amplify the inflammatory response.^1,5,8,17^ Concurrently, vascular smooth muscle cells proliferate and undergo phenotypic modulation from a differentiated phenotype involved in collagen synthesis to a dedifferentiated proinflammatory phenotype.^21–24^ Collectively, these changes, in conjunction with apoptotic cell death and release of matrix metalloproteinases, may lead to remodeling and rupture of the aneurysm wall.^5,25–28^

Several therapies that target the inflammatory response have been investigated in animal models of cerebral aneurysms.^5^ Promising results were reported with NF‐kB inhibitors,^2^ statins,^29^ matrix metalloproteinases inhibitors,^25^ free‐radical scavengers,^30^ and inhibitors of mast cell degranulation.^27^ Although these therapies appear to attenuate aneurysm formation and progression in experimental animals, it is not clear whether they protect against rupture of aneurysms. Furthermore, extrapolation of the results of studies in experimental animals to humans may not be appropriate.^31^

It is important to develop noninvasive therapies for intracranial aneurysms, because there are significant limitations in current therapeutic options, namely, microsurgical clipping and endovascular treatment. The natural history of intracranial aneurysms should be balanced against the morbidity associated with these procedures.^32–33^ This small study suggests, in accordance with previous reports,^34–35^ that a low dose of ASA treatment may be a promising agent for reducing vascular inflammation.

### Limitations

The small number of patients enrolled is a limitation of this study. Despite this limitation, sufficient data were obtained to reveal consistent patterns of labeling and to demonstrate the anti‐inflammatory effects of ASA in aneurysm walls. Also, the small size of the aneurysm dome tissues collected during surgery precluded examination of expression of additional inflammatory cells and molecules. This experiment was conducted in patients with “low‐risk” aneurysms, and this approach may not be appropriate in patients harboring aneurysms that are at high risk of rupture.

## Conclusions

This study provides preliminary radiographic and histological data showing that a low dose of ASA may attenuate inflammation in the walls of human cerebral aneurysms. ASA may therefore be a promising agent to target the inflammatory response in the walls of aneurysms. These observations may be related to our previous findings that ASA use decreases the risk of aneurysm rupture. Further studies with larger patient populations are warranted to evaluate these preliminary conclusions.
